# Access to Mental Health Care for Patients With Neurological Conditions

**DOI:** 10.1192/bjo.2026.11642

**Published:** 2026-06-30

**Authors:** Soracha Healy, Dhruvi Patel, Ellen McCloy-Smith, Faye Stanage, Akshay Nair

**Affiliations:** 1 South West London and St George's NHS Mental Health Trust, London, United Kingdom; 2 City St George's University of London, London, United Kingdom; 3 St George's Hospital, London, United Kingdom

## Abstract

**Aims:**

- To understand the perspectives of mental health professionals on the management of neuropsychiatric patients and their access to mental health services
- To identify key themes that could help improve collaboration between neurology, neuropsychiatry and broader mental health services
- To enhance mental health care for patients with neurological conditions

**Methods:**

An online survey was designed and sent to multidisciplinary team members in working age and older adult services in South West London and St George’s (SWLSTG) and Surrey and Borders NHS Trusts (SABP) which collected both qualitative and quantitative data.

**Results:**

There were 29 respondents to the survey with 21 from SWLSTG and 8 from SABP. The majority of respondents were doctors with the largest group being consultants (10) and resident doctors (8) followed by psychologists (5), nurses (3) and other (3). 79.3% of respondents have had 5 or more years of experience working in mental health services and most work in community teams (38.0%) and Liaison (28.0%).

93.1% of clinicians have cared for someone with co-occurring mental health conditions and neurological disorders in the last 12 months. 96.6% of respondents felt confident in assessing the mental health of patients in their day to day practice. However, confidence reduced to 55.2% when assessing the mental health of someone with a neurological disorder.

Crucially, 79.3% of respondents do not feel the service they work in is well designed to look after patients with neurological conditions and co-existing mental health problems. While only 20.7% agree they have access to specialised resources or support for managing such patients, 69.0% do feel supported by their colleagues and seniors in these circumstances.

The majority of respondents think it is harder for someone with a co-occurring mental health issue and neurological condition to access their service compared to those with common or severe mental health conditions. Over 75% of respondents do not think that routine general adult mental health services are well equipped to manage patients with neurological disorders and do not feel there is enough input from neurology services.

**Conclusion:**

There is a gap in service delivery and clinician confidence for patients with neurological conditions who require mental health care. Better access to neuropsychiatric services for advice and co-working opportunities were identified as key areas for improvement. The next steps will involve liaising with local services to create a network of secondary care and third sector organisations and consider the development of guidelines.

